# CRISPR-Cas9 Screen Identifies DYRK1A as a Target for Radiotherapy Sensitization in Pancreatic Cancer

**DOI:** 10.3390/cancers14020326

**Published:** 2022-01-10

**Authors:** Bin Lan, Siyuan Zeng, Shuman Zhang, Xiaofan Ren, Yuming Xing, Isabella Kutschick, Susanne Pfeffer, Benjamin Frey, Nathalie Britzen-Laurent, Robert Grützmann, Nils Cordes, Christian Pilarsky

**Affiliations:** 1 Department of Surgery, Universitätsklinikum Erlangen, Friedrich-Alexander Universität Erlangen-Nürnberg (FAU), 91054 Erlangen, Germany; binlan1991@163.com (B.L.); siyuanzeng2021@163.com (S.Z.); zhang_shu_man@163.com (S.Z.); renxiaofan1013@gmail.com (X.R.); yumingxingjennifer@gmail.com (Y.X.); isabella.kutschick@uk-erlangen.de (I.K.); susanne.pfeffer@uk-erlangen.de (S.P.); nathalie.britzen-laurent@uk-erlangen.de (N.B.-L.); robert.gruetzmann@uk-erlangen.de (R.G.); 2 Translational Radiobiology, Department of Radiation Oncology, Universitätsklinikum Erlangen, Friedrich-Alexander Universität Erlangen-Nürnberg (FAU), 91054 Erlangen, Germany; benjamin.frey@uk-erlangen.de; 3 OncoRay-National Center for Radiation Research in Oncology, Faculty of Medicine Carl Gustav Carus, Technische Universität Dresden, 01307 Dresden, Germany; nils.cordes@oncoray.de; 4 Helmholtz-Zentrum Dresden-Rossendorf, Institute of Radiooncology-OncoRay, 01328 Dresden, Germany; 5 German Cancer Consortium, Partner Site Dresden: German Cancer Research Center, 69120 Heidelberg, Germany; 6 Department of Radiotherapy and Radiation Oncology, University Hospital Carl Gustav Carus, Technische Universität Dresden, 01307 Dresden, Germany

**Keywords:** pancreatic cancer, CRISPR-Cas9, radiotherapy, radioresistance, DYRK1A, Harmine, DNA damage repair, DNA double-strand break

## Abstract

**Simple Summary:**

Pancreatic cancer is the fourth leading cause of cancer-related death in Western countries. Although several therapeutic strategies have been developed for pancreatic cancer, radiation therapy has not yet yielded satisfactory results. Unraveling the mechanism of radioresistance in pancreatic cancer and developing new therapeutic targets has become a major challenge. Therefore, we applied kinome-wide CRISPR-Cas9 loss-of-function screening combined with the 3D cell culture method and identified DYRK1A as a sensitive target for radiotherapy. Additionally, we confirmed that DYRK1A-targeted inhibitors could enhance the efficacy of radiotherapy. Our results further support the use of CRISPR-Cas9 screening to identify novel therapeutic targets and develop new strategies to enhance radiotherapy efficacy in pancreatic cancer.

**Abstract:**

Although radiation therapy has recently made great advances in cancer treatment, the majority of patients diagnosed with pancreatic cancer (PC) cannot achieve satisfactory outcomes due to intrinsic and acquired radioresistance. Identifying the molecular mechanisms that impair the efficacy of radiotherapy and targeting these pathways are essential to improve the radiation response of PC patients. Our goal is to identify sensitive targets for pancreatic cancer radiotherapy (RT) using the kinome-wide CRISPR-Cas9 loss-of-function screen and enhance the therapeutic effect through the development and application of targeted inhibitors combined with radiotherapy. We transduced pancreatic cancer cells with a protein kinase library; 2D and 3D library cells were irradiated daily with a single dose of up to 2 Gy for 4 weeks for a total of 40 Gy using an X-ray generator. Sufficient DNA was collected for next-generation deep sequencing to identify candidate genes. In this study, we identified several cell cycle checkpoint kinases and DNA damage related kinases in 2D- and 3D-cultivated cells, including DYRK1A, whose loss of function sensitizes cells to radiotherapy. Additionally, we demonstrated that the harmine-targeted suppression of DYRK1A used in conjunction with radiotherapy increases DNA double-strand breaks (DSBs) and impairs homologous repair (HR), resulting in more cancer cell death. Our results support the use of CRISPR-Cas9 screening to identify new therapeutic targets, develop radiosensitizers, and provide novel strategies for overcoming the tolerance of pancreatic cancer to radiotherapy.

## 1. Introduction

The most frequent form of pancreatic cancer (PC), pancreatic ductal adenocarcinoma (PDAC), is one of the most common causes of cancer death worldwide [1]. With few symptoms showing before the disease reaches its advanced stage and its extremely aggressive nature, PDAC prognosis at the time of diagnosis is often dim. Despite the advancements made in the detection and management of PDAC, the 5-year survival rate still stands at only 9% [1,2]. Resection, the only curative therapeutic option for PDAC, is effective in approximately 15–20% of cases and has a limited effect, with just 20% of resected patients living more than five years [3]. To improve survival, chemotherapy (CT) and radiotherapy (RT) are used in combination with resection or as the sole treatment for 80–85% of PC patients with unresectable tumors [4]. Unfortunately, despite the development of Stereotactic Body Radiation Therapy (SBRT) and other therapy approaches, RT does not play a decisive role in the treatment of PDAC and is usually only mildly successful in a few cases of both resectable and unresectable tumors [2,4,5,6]. Although preoperative (neoadjuvant) chemoradiotherapy is the standard of care for many other cancers, preoperative (neoadjuvant) chemoradiotherapy for patients with resectable or borderline resectable PDAC has not been shown to have a significant overall survival benefit [7]. Recent studies have shown that preoperative chemoradiotherapy increases the R0 resection rate, decreases the lymph node positivity rate, and diminishes local and distant recurrence rates by inducing the downstaging of the tumor [8,9].

Radiotherapy for pancreatic cancer often fails due to radiation toxicity and radioresistance. Radioresistance in patients with PC, either inherent or acquired, is mediated by DNA damage repair, cell cycle checkpoints, tumor stem cells, tumor microenvironment, and other factors [10]. Radioresistance remains a significant barrier impeding the broad adoption of radiotherapy for pancreatic cancer treatment, and the development of effective sensitizers or strategies to reverse radioresistance represents a promising area of research.

In tumor cells, ataxia–telangiectasia mutated (ATM) and ATM- and Rad3 Related (ATR) are major members of DNA damage checkpoints and are activated by different types of DNA damage. Their downstream factors are checkpoint kinase 2 (CHK2) and checkpoint kinase 1 (CHK1), respectively [11]. Both kinases are involved in the regulation of the DNA damage response by initiating cell cycle checkpoint control and activating the appropriate DNA repair pathways [12,13,14]. A variety of radiosensitization approaches targeting DNA damage and DNA repair have been attempted in PC [15,16,17]. Although most of these approaches have demonstrated promising outcomes in preclinical studies performed in vitro and in vivo, the majority eventually failed to deliver meaningful radiosensitization in clinical trials owing to their severe side effects and limited effectiveness [2,5]. As a result, it is important to discover and understand the underlying processes causing this treatment to fail in order to gain a better understanding of how to build effective radiosensitization regimens.

The development of CRISPR-Cas9 screening approaches in recent years has enabled the focused discovery of tumor treatment resistance mechanisms, paving the way for the creation of novel targeted therapies. In this study, we aimed to use a genome-wide CRISPR-Cas9 loss-of-function screen to identify sensitive targets for radiotherapy in pancreatic cancer and enhance the efficacy of radiotherapy by developing and applying targeted inhibitors in combination with radiotherapy.

## 2. Materials and Methods

### 2.1. Cell Culture and Reagents

In this study, the human pancreatic cancer cell line MIA PaCa-2 (ATCC, Cat# CRM-CRL-1420, RRID: CVCL_0428) and the murine pancreatic cancer cell line TB32047 were used. MIA PaCa-2 cells were purchased from American Type Culture Collection (ATCC, Manassas, VA, USA). The primary murine pancreatic cancer cell line TB32047 was thankfully provided by David Tuveson (Cold Spring Harbor Laboratory, Cold Spring Harbor, NY, USA). All cell lines were grown in monolayer culture in a humidified atmosphere containing 5% CO_2_ at 37 °C. TB32047 cells were cultured in DMEM medium (Cat# 30966-021, Gibco) supplemented with 10% FBS. MIA PaCa-2 cells cultured in DMEM medium with 10% FBS and 2% horse serum (Cat# 16050-130, Gibco). HEK293TN (Cat# LV900A-1-GVO-SBI, RRID:CVCL_UL49, BioCat, Heidelberg, Germany) cells were obtained from BioCat and cultured in DMEM supplemented with 10% heat-inactivated FBS. Murine pancreatic ductal 3D/spheroid culture was described previously (15, 16). In brief, TB32047 cells were digested and further mixed with Matrigel (BD Bioscience, San Jose, CA, USA) and cultured in complete feeding medium (AdDMEM/F12 medium supplemented with HEPES (1×, Invitrogen, Waltham, MA, USA), Glutamax (1×, Invitrogen), penicillin/streptomycin (1×, Invitrogen), B27 (1×, Invitrogen), Primocin (1 mg/mL, InvivoGen, San Diego, CA, USA), N-acetyl-L-cysteine (1 mM, Sigma, St. Louis, MO, USA), Wnt3a-conditioned medium (50% *v*/*v*), RSPO1-conditioned medium (10% *v*/*v*, Calvin Kuo, Stanford, CA, USA), Noggin conditioned medium (10% *v*/*v*) or recombinant protein (0.1 μg/mL, Peprotech, Rocky Hill, NJ, USA), epidermal growth factor (EGF, 50 ng/mL, Peprotech), Gastrin (10 nM, Sigma), fibroblast growth factor 10 (FGF10, 100 ng/mL, Prepotech), Nicotinamide (10 mM, Sigma) and A83-01 (0.5 μM, Tocris, Bristol, UK). All cells were harvested by 0.25% Trypsin-EDTA (Ethylenediaminetetraacetic acid) (Cat# 25200-072, Gibco). Cells were authenticated by DNA fingerprinting using highly polymorphic short tandem repeat (STR) analysis, and mycoplasma detection was regularly conducted to confirm the absence of contamination.

The DYRK1A targeted inhibitor Harmine (Cat# S3817) was purchased from Selleck Chemicals GmbH (Houston, TX, USA).

### 2.2. The sgRNA Library and Lentivirus Production

The pooled-sgRNA library (Mouse Brie kinome pooled library) targeting murine kinome was a gift from John Doench and David Root [18] (RRID: Addgene_75316, Addgene, Cambridge, MA, USA) and modified for the inclusion of pancreatic cancer-related genes by our lab (supplemental data). The resulting library contained 3548 sgRNAs for 924 murine genes. The lentivirus was produced as described previously [19]. Briefly, three T175 flasks of HEK293TN cells were plated at 70% confluence and incubated overnight. For each flask, 13.8 μg of pooled-sgRNA library, 9.2 μg of pMDLg/pRRE (Cat# 12251, Addgene), 4.6 μg of pRSV-REV (Cat# 12253, Addgene), 4.6 μg of pMD2.G (Cat# 12259, Addgene), 64.4 μL P3000 Enhancer Reagent, and 129 μL of Lipofectamine™ 3000 diluted in OptiMEM (Cat# 31985070, Gibco) were mixed and added to the HEK293TN cells. pMDLg/pRRE (Addgene, RRID: Addgene_12251), pRSV-REV (Addgene, RRID: Addgene_12253), and pMD2.G (Addgene, RRID: Addgene_12259) were gifts from Didier Trono [20]. The medium was changed 6 h post transfection and the virus was collected by filtering through a 0.45 μm strainer 24 h later.

### 2.3. CRISPR/Cas9 Knockout Screening

The CRISPR screening was performed as described previously [21]. Briefly, 1.8 million TB32047 cells were transduced in triplicate with the sgRNA lentivirus library (TBlib1, TBlib2, TBlib3). Cells were then selected with puromycin (10 μg/mL) for 3 days. Ten million transfected cells were harvested at the baseline (Day 0). The remaining surviving cells were divided into two groups, 2D and 3D screening groups. In the experimental group, 2D and 3D library cells were irradiated daily with a single dose of up to 2 Gy for 4 weeks for a total of 40 Gy using an X-ray generator (120  kV, 25 mA, GE Inspection Technologies, Ahrensburg, Germany), while unirradiated cells were consecutively cultured for 4 weeks as a control group. In 2D functional screening, there was at least 300-fold coverage for each sgRNA, and in 3D screening there was at least 80-fold coverage for all sgRNAs to guarantee a sufficient number of sgRNAs. At least 10 million cells for each group were collected for DNA sequencing at the end of the screening.

### 2.4. Genomic DNA Sequencing and Data Analysis

The isolation of genomic DNA (gDNA) was performed with a NucleoSpin Blood L kit (Cat# 740954.20, Macherey-Nagel, Düren, Germany) and followed by PCR procedure to amplify sgRNAs. In order to obtain 300-fold coverage, 10 µg of DNA was utilized in the PCR process. For each sample, the Q5 master mix (Cat# M0494S, Biolabs, Beverly, MA, USA) was used to conduct two independent 100 µL reactions with 5 µg of genomic DNA in each reaction and then the amplicons were concatenated. The following primers were used and synthesized by Eurofins Genomics: P5, 5′-ACACTCTTTCCCTACACGACGCTCTTCCGATCTNNNNNTCTTGTGGAAAGGACGAAACACCG-3′, and P7, 5′-TCTACTATTCTTTCCCCTGCACTGT-3′. PCR amplicons generated during the PCR reactions were purified and sequenced using a NovaSeq 6000. The number of reads for each sgRNA was measured and normalized to the total number of reads for all sgRNAs using the MAGeCK-VISPR program, as described previously [22].

### 2.5. CRISPR-Cas9 Gene Editing

DYRK1A was knocked out in TB32047 and MIA PaCa-2 cell lines with the CRISPR-Cas9 gene editing system, as described before [23]. The following sgRNAs were used and synthesized by Eurofins Genomics:

Mm_Dyrk1a_sg1-forward (5′-CACCGTAATAGGAGTACAAACCACC-3′), Mm_Dyrk1a_sg1-reverse (5′-AAACGGTGGTTTGTACTCCTATTAC-3′), Mm_Dyrk1a_sg4-forward (5′-CACCGTCATTGGCACCACTGAACAG-3′), Mm_Dyrk1a_sg4-reverse (5′-AAACCTGTTCAGTGGTGCCAATGAC-3′), Mm_non-targeting control-forward (5′-CACCGACGCGAAGTGTCGCAGAGTG-3′), Mm_non-targeting control-reverse (5′-AAACACGCGAAGTGTCGCAGAGTG-3′), Hs_DYRK1A_sg1-forward (5′-CACCGTGAGAAACACCAATTTCCGA-3′), Hs_DYRK1A_sg1-reverse (5′-AAACTCGGAAATTGGTGTTTCTCAC-3′), Hs_DYRK1A_sg4-forward (5′-CACCGAACGGAAGGTTTACAATGA-3′), Hs_DYRK1A_sg4-reverse (5′-AAACTCATTGTAAACCTTCCGTTC-3′), Hs_non-targeting control-forward (5′-CACCGGAACTCAACCAGAGGGCCAA-3′), and Hs_non-targeting control-reverse (5′-AAACTTGGCCCTCTGGTTGAGTTC-3′).

Knockout was performed using the pSpCas9(BB)-2A-Puro (PX459) V2.0 vector, which was a gift from Feng Zhang (Addgene, RRID: Addgene_62988) [19]. The ligated vector was inserted into Endura Electrocompetent Cells (Cat# 60242-1, Lucigen, Middleton, WI, USA). Cells were transfected with the DYRK1A knock-out plasmid by lipofectamine transfection reagent for 24 h and followed by puromycin selection for three days. Limited dilution was performed by plating one single cell per well of knockdown cells in a 96-well plate to select single clones. After growth, Western blotting was performed to verify the knockout. The wild-type cells were named WT, and the single clone cells transfected with non-targeting control sgRNAs and transfected with DYRK1A sgRNAs were named NC and KO cells, respectively.

### 2.6. Clonogenic Assay

In brief, cells (1000–2000 cells/well) were plated in triplicate on a 6-well plate, incubated for 24 h, irradiated with X-rays and cultured for further 7 to 12 days. Surviving colonies were fixed with formalin and stained with 0.5% crystal violet. Colonies containing more than 50 cells were counted. The relative colony-forming efficiency was determined by normalizing the colony numbers to the untreated control.

### 2.7. Alkaline Comet Assay

The alkaline comet assay was performed using the CometAssay HT Kit (Cat# 4252-050-K, Trevigen, Gaithersburg, MD, USA ) according to the manufacturer’s instructions. Briefly, cells were mixed with molten low-melt agarose (LMAgarose), then the mixture was immediately evenly dispersed onto a CometSlide. The slides were then placed at 4 °C in the dark for 30 min in a high-humidity environment. The cells were then lysed overnight with lysis buffer. After lysis, the slides were rinsed in distilled water and immersed in alkaline electrophoresis buffer for 30 min before electrophoresis. An electric field (1 V/cm) was applied to the cells for 35 min at 4 °C, and the cells were stained with GelRed Nucleic Acid Stain (Cat# 41003, LINARIS GmbH, Dossenheim, Germany) for 20 min in the dark and photographed using a Evos FL Auto 2 imaging system (Invitrogen, AMAFD2000). The results were analyzed using the CASP v1.2.3beta2 software (Wroclaw, Poland) [24].

### 2.8. Western Blot

Western blot analysis was performed as described [23]. In brief, cells were lysed in RIPA buffer (Cat# 89900, Thermo Fisher Scientific, Waltham, MA, USA) containing protease and phosphatase Inhibitor (Cat# 78442, Thermo Fisher Scientific) and the protein concentration was determined using the BCA Protein Assay Kit (Cat# 23250, Thermo Fisher Scientific). Equal amounts of total protein were separated on 4–12% NUPAGE Bis-Tris gels (Cat# NP0322BOX; Thermo Fisher Scientific) using the Mini Gel Tank chamber system (Invitrogen) and proteins were transferred to a nitrocellulose membrane (Cat# GE10600003; Sigma-aldrich, St. Louis, MO, USA). After blocking with 5% milk, the following primary antibodies were used and incubated overnight at 4 °C: Chk1 (Cell Signaling Technology, Danvers, MA, USA, Cat# 2360, RRID:AB_2080320), phospho-Chk1 (Ser345) (CST, Cat# 2341, RRID:AB_330023), phospho-Chk1 (Ser296) (CST, Cat# 2349, RRID:AB_2080323), ATM (CST, Cat# 2873, RRID:AB_2062659), phospho-ATM (Ser1981) (Santa Cruz Biotechnology, Santa Cruz, CA, USA, Cat# sc-47739, RRID:AB_781524), Chk2 (CST, Cat# 2662, RRID:AB_2080793), hosphor-Chk2 (Thr68) (Abcam, Cambridge, MA, USA, Cat# ab3501, RRID:AB_449196), Ku70 (CST, Cat# 4588, RRID:AB_11179211), Rad51(CST, Cat# 8875, RRID:AB_2721109), and phospho-Histone H2A.X (Ser139) (CST, Cat# 2577, RRID:AB_2118010). GAPDH (CST, Cat# 5174, RRID:AB_10622025) and Vinculin (CST, Cat# 18799, RRID:AB_2714181) served as the loading controls. HRP-linked anti-rabbit IgG (CST, Cat# 7074, RRID:AB_2099233) and HRP-linked anti-mouse IgG (CST, Cat# 7076, RRID:AB_330924) were used as the secondary antibody. The quantification of signals was performed by an Amersham Imager 600 (Pittsburgh, PA, USA) with the SignalFire™ ECL Reagent (Cat# 6883S, CST).

### 2.9. Immunofluorescence

For immunofluorescence, cells were seeded on poly-lysine-treated cover slides, fixed with 4% formalin for 15 min at room temperature, and permeabilized with 0.1% Triton X-100 for 30 min. Fixed cells were then blocked with 10% goat normal serum and incubated with phospho-Histone H2A.X (Ser139) overnight at 4 °C. The next day incubated with Alexa Fluor 488 goat anti-Rabbit IgG (Thermo Fisher Scientific, Cat# A-11034, RRID:AB_2576217) for 1 h at room temperature. Nuclei were stained with DAPI (Cat# 62248, Life Technologies, Gaithersburg, MD, USA) for 10 min. Images were acquired on a Leica LSM microscope TCS SP8 (Leica Microsystems CMS GmbH, Mannheim, Germany) and processed using the Leica Application Suite X v2.0.1.14392 software.

### 2.10. Apoptosis Assay

An apoptosis assay was performed with the FITC Annexin V Apoptosis Detection Kit I (Cat# 556547, BD Pharmingen, San Diego, CA, USA) according to the manufacturer’s instructions. Briefly, cells in the supernatant and adherent to plates were collected, washed with PBS, resuspended in binding buffer, and stained with annexin V-FITC and PI for 15 min at room temperature. Then, they were analyzed on a BD Biosciences LSRII flow cytometer. The flow cytometry results were analyzed using the FlowJo v10.8 Software (BD Life Sciences, Ashland, OR, USA).

### 2.11. Statistical Analysis

Statistical analyses were performed using GraphPad Prism 8.0 (GraphPad Software Inc., La Jolla, CA, USA). In all studies, data represent biological replicates (n) and are depicted as means ± SDs, as indicated in the figure legends. Statistical significance was determined by the 2-tailed unpaired Student’s *t* test. *p*-values are reported in the graphs. *, *p* < 0.05; **, *p* < 0.01; ***, *p* < 0.001; and ****, *p* < 0.0001. ns. not significant. In all analyses, *p* < 0.05 was considered statistically significant.

## 3. Results

### 3.1. CRISPR-Cas9 Loss-of-Function Screen Identifies DYRK1A as Candidate for Radiotherapy Resistance

**Figure 1:**
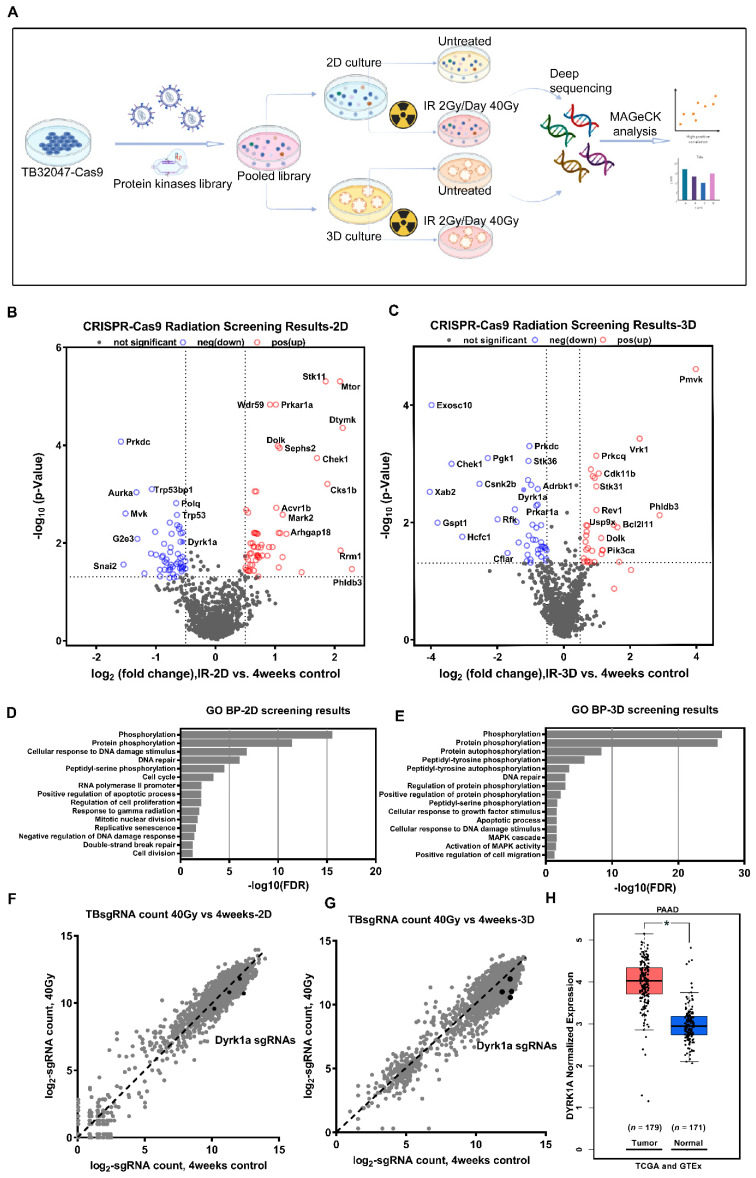
CRISPR-Cas9 loss-of-function screen identifies DYRK1A as a candidate for radiotherapy resistance. (**A**) Schematic diagram of the experimental procedure for CRISPR-Cas9 loss-of-function screen. (**B**,**C**) Volcano plots show the -log normalized *p*-value and log_2_ fold change of 2D (**B**) and 3D (**C**) radiation screening results compared to the 4-week control, with significant genes in the negative selection highlighted in blue and positive selection highlighted in red, respectively. (**D**,**E**) Gene Ontology (GO) terms analyzed the biological processes enriched by 2D (**D**) and 3D (**E**) negative selection candidate genes. (**F**,**G**) Scatter plots show the log_2_-normalized counts changes of each sgRNA of DYRK1A in 2D (**F**) and 3D (**G**) screening. (**H**) Box plots of DYRK1A expression in pancreatic cancer and normal pancreatic tissues were analyzed using the TCGA (PAAD) and GTEx (pancreas) databases. *, *p* < 0.05.

In order to investigate the mechanism of pancreatic cancer radioresistance and to discover novel therapeutic targets, we combined the latest CRISPR-Cas9 loss-of-function screen and 3D culture technology to perform an extensive CRISPR-Cas9 screen in the pancreatic cancer cell line TB32047 derived from the KPC mouse model (Figure 1A).

The concurrent prioritization of candidate genes based on commonality among sgRNAs resulted in a significant reduction in most of the sgRNAs targeting the same gene (*p* < 0.05) to minimize off-target effects, and we ultimately identified 68 and 50 kinase hits in the 2D and 3D screens, respectively (Figure 1B,C; Supplementary Table S1). Furthermore, we used Gene Ontology (GO) term biological process analysis (DAVID Bioinformatics Resources 6.8) to enrich the cellular processes for significant candidate genes in 2D and 3D screening results. The annotation of these genes indicated that they belong to several functional categories, phosphorylation, protein phosphorylation and autophosphorylation, DNA repair, cell cycle, apoptotic processes, etc. (Figure 1D,E). Interestingly, the same cellular processes enriched in the 2D and 3D screens included phosphorylation, protein phosphorylation, cellular response to DNA damage stimulus, DNA repair, and peptidyl-serine phosphorylation, supporting the biological plausibility of our results. Only two genes, Prkdc and Dyrk1a, were found among the 20 most significant candidates from both the 2D and 3D screenings (Supplementary Table S1).  Protein kinase, DNA-activated, catalytic subunit (PRKDC), alternatively referred to as DNA-PKcs, functions as a molecular sensor of DNA damage, which engaged in the non-homologous end joining (NHEJ) pathway for a DNA double-strand break (DSB) repair process [25]. All four sgRNAs targeting the second candidate, DYRK1A (dual-specificity tyrosine phosphorylation-regulated kinase 1A), were significantly reduced in both 2D and 3D screens (Figure 1F,G). The TCGA (PAAD) and GTEx (Pancreas) databases indicated that the DYRK1A expression level was significantly higher in pancreatic cancer than in the corresponding normal pancreas tissue (Figure 1H). Moreover, the role of DYRK1A in the radioresistance of pancreatic cancer has not been reported. Therefore, we focused on the role of DYRK1A in the radiotherapy of pancreatic cancer in the following experiments.

### 3.2. DYRK1A Knockout Enhances Radiotherapy Efficacy in Pancreatic Cancer Cells

**Figure 2:**
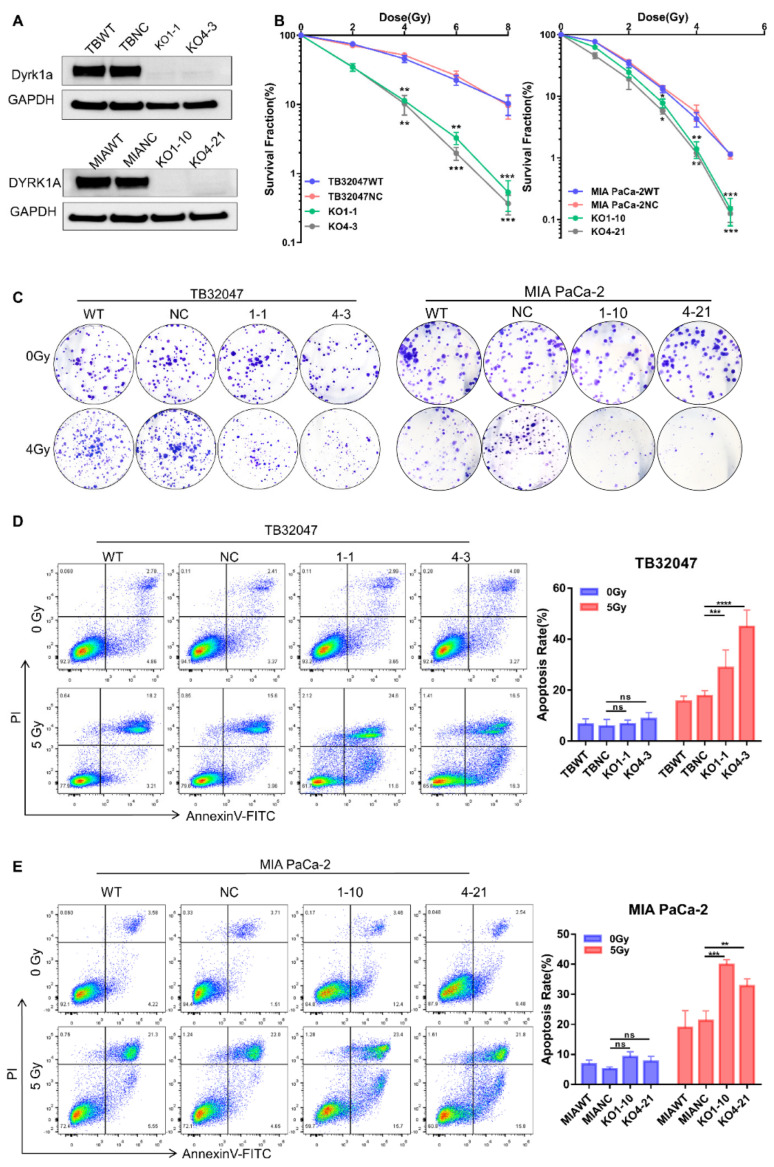
DYRK1A knockout enhances radiotherapy efficacy in pancreatic cancer. (**A**) Western blot of TB32047 and MIA PaCa-2 cells transfected with DYRK1A sgRANs or non-targeting control. (**B**) A clonogenic assay of TB32047 (**left**) and MIA PaCa-2 (**right**) cells with DYRK1A KO and control sgRNA cells was performed after irradiation with different doses of X-rays. The colony numbers were counted and normalized. Data are presented as means of three independent experiments (*n* = 3). *, *p* < 0.05; **, *p* < 0.01; *** and *p* < 0.001 by 2-tailed unpaired Student’s *t* test. (**C**) TB32047 (**left**) and MIA PaCa-2 (**right**) single clones and control cells were treated with different doses of radiation followed by clonogenic assays. Representative images of three independent experiments (*n* = 3) are shown. (**D,E**) Flow cytometry-based apoptosis analysis of TB32047 (**D**) and MIA PaCa-2 (**E**) control and DYRK1A KO single clones irradiated with 5 Gy X-rays after 24 h. Representative images of three independent experiments (*n* = 3) and statistical analysis are shown. **, *p* < 0.01; ***, *p* < 0.001 and ****, *p* < 0.0001 by 2-tailed unpaired Student’s *t* test. ns. not significant.

To demonstrate that DYRK1A may be exploited as a target for improved radiation therapy in pancreatic cancer, we used two separate sgRNAs to knock out the DYRK1A gene in TB32047 and MIA PaCa-2 cells and generated totally knocked out single clone cells (Figure 2A). Clonogenic assays showed a dramatic decrease in the colony number DYRK1A-knock-out (KO) single clones after irradiation (IR) compared to cell clones transduced with non-targeting control sgRNA (NC) or wild-type (WT) cells, indicating a higher sensitivity to X-rays in the absence of DYRK1A (Figure 2B,C).

In the absence of radiation treatment, flow cytometry analysis indicated that there was no significant difference in early and late apoptosis in DYRK1A-deleted cells compared to parental and NC cells. By contrast, when DYRK1A KO cells were irradiated with 5 Gy X-rays for 24 h, both early and late apoptosis were considerably enhanced (Figure 2D,E). These results indicated that deleting DYRK1A impairs colony formation ability and increases cell death during radiation treatment.

### 3.3. DYRK1A Knockout Increases DNA Damage and DNA Double-Strand Breaks in Pancreatic Cancer Cells after Radiotherapy

**Figure 3:**
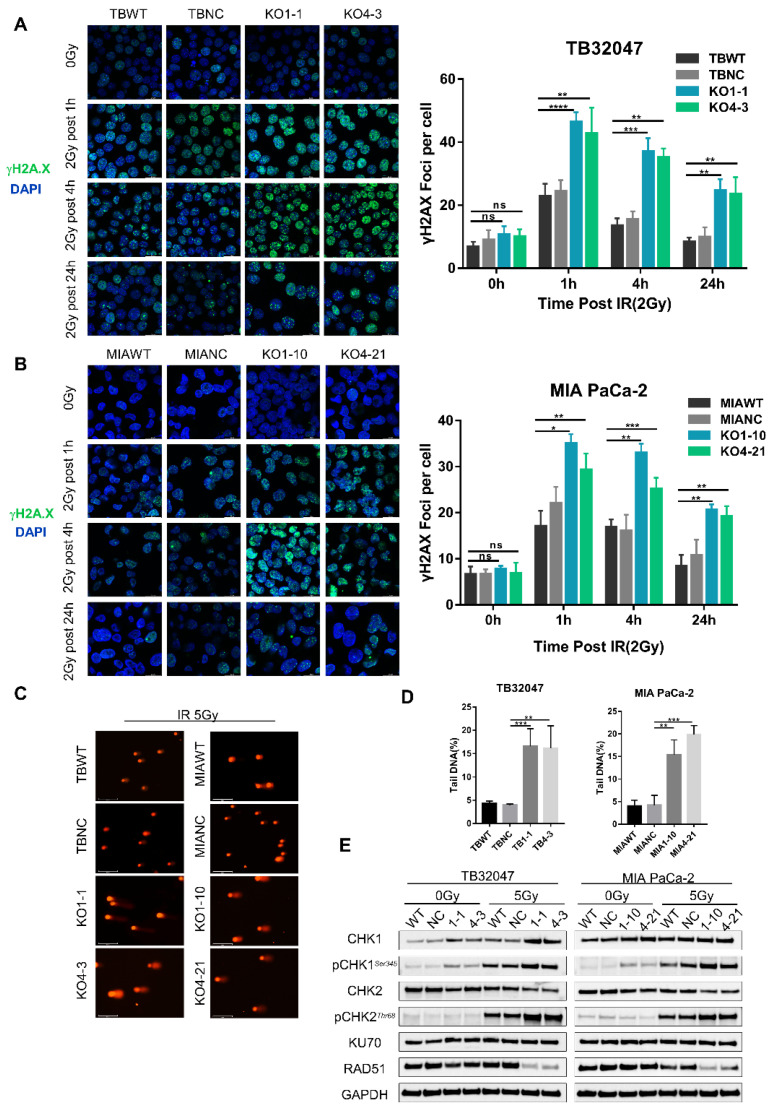
DYRK1A knockout increases DNA damage and DNA double-strand breaks in pancreatic cancer cells after radiotherapy. (**A**,**B**) Immunofluorescence of TB32047 (**A**) and MIA PaCa-2 (**B**) control or DYRK1A KO single clones at different time points with 2Gy irradiation. Representative images of three independent experiments (*n* = 3) and statistical analysis are shown. (Blue-DAPI, Green-γH2A.X. Scale bar: white, 20 μm). *, *p* < 0.05; **, *p* < 0.01; ***, *p* < 0.001 and ****, *p* < 0.0001 by 2-tailed unpaired Student’s *t* test. ns. not significant. (**C**) Alkaline comet assay of TB32047 and MIA PaCa-2 knockout single cells and control cells after irradiation with 5 Gy X-rays. Representative images of three independent experiments (*n* = 3) are shown (scale bar: white, 125 μm). (**D**) CASP software analysis of the percentage of DNA content in the comet tail of the alkaline comet experiment. A minimum of 50 cells were statistically analyzed. **, *p* < 0.01 and ***, *p* < 0.001 by 2-tailed unpaired Student’s *t* test. (**E**) Western blot of TB32047 and MIA PaCa-2 control or DYRK1A KO single clone cells with 0 Gy or 5 Gy irradiation after 24 h.

To examine the potential mechanisms leading to increased apoptosis under radiotherapy in DYRK1A-KO cells, we first investigated the levels of DNA damage using phosphorylated histone H2A.X as a marker of stalled replication forks and DSB [26,27]. We followed the kinetics of DSB formation and repair in TB32047 and MIA Paca-2 cells after a single dose of X-rays (2-Gy). Cells were harvested after 1, 4, and 24 h before performing the immunofluorescence staining of phosphorylated (γ) H2A.X and counting counted positive γH2A.X foci. The number of γH2A.X foci increased dramatically in KO cells after radiation exposure and peaked at 1 h post-irradiation before gradually decreasing, likely due to the activation of DNA damage repair (DDR) mechanisms following radiation. Although radiation also caused a short-term increase in γH2A.X foci in control cells, the magnitude of the increase was significantly smaller than that of knockdown cells at the different time points. Twenty-four hours after irradiation, the number of γH2A.X foci of wild-type cells was reduced to baseline levels, while more DSBs remained unrepaired in KO cells in both TB32047 and MIA PaCa-2 cells (Figure 3A,B). In the absence of radiation, DYRK1A single clones did not exhibit an increase in γH2A.X-positive cells and γH2A.X foci compared to the DYRK1A wild-type and NC cells (Figure 3A,B). This is also consistent with the results of apoptosis in unirradiated cells (Figure 2D,E), demonstrating that the deletion of DYRK1A does not induce H2A.X phosphorylation or apoptosis.

To determine whether the strong increase in γH2A.X foci in DYRK1A-KO cells after irradiation was due to an increase in DNA DSBs, we used the alkaline comet assay, which is a sensitive method for monitoring DSBs. We detected that the irradiation of X-rays with 5-Gy increased the comet tail moment significantly in DYRK1A KO cells (Figure 3C,D). Together with the increased γH2A.X foci number, this suggested enhanced DSB formation and/or replication fork collapse in these cells. Consistent with the presence of γH2A.X in DYRK1A KO cells, we observed the activation of CHK1 and CHK2 phosphorylation (Figure 3E), which were key molecules in transducing DNA damage signals. In contrast, the radiation treatment of DYRK1A KO PC cells resulted in decreased levels of RAD51, a key protein for HR repair [28], but not of Ku70, an essential protein for NHEJ [29] (Figure 3E). These results indicate that DYRK1A deficiency enhances DNA damage and impairs DNA damage homologous repair after radiotherapy.

### 3.4. Targeted Inhibition of DYRK1A Promotes Radiosensitivity in Pancreatic Cancer

**Figure 4:**
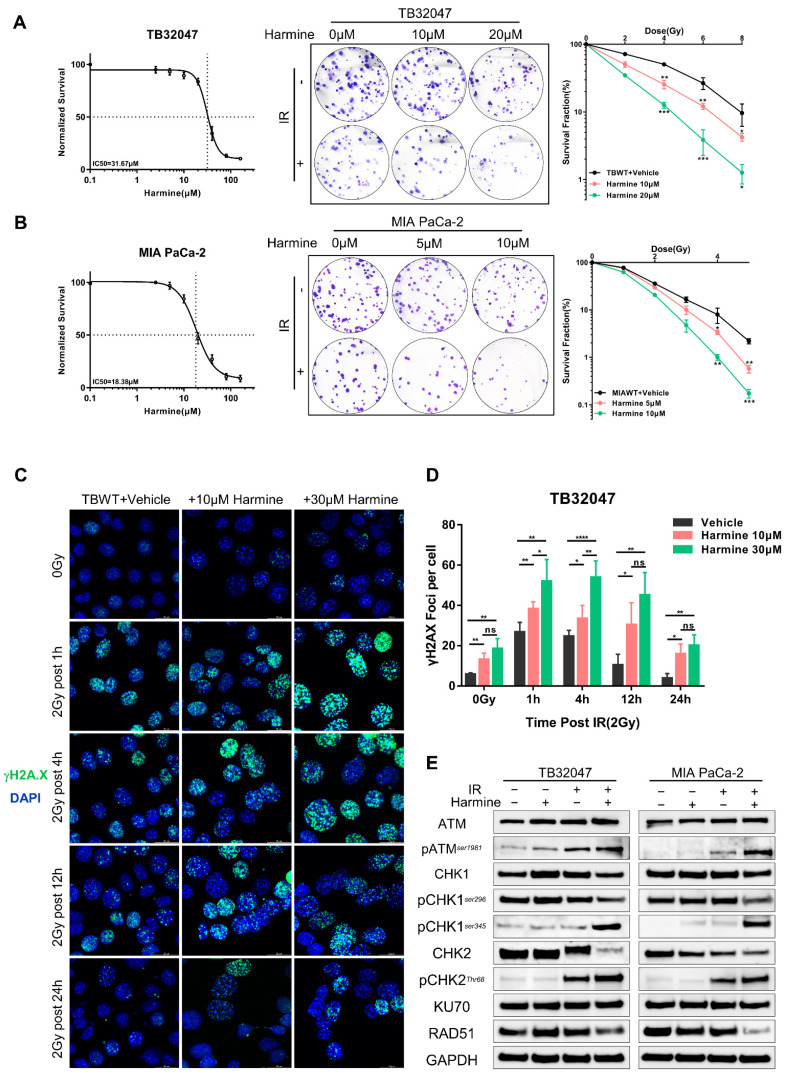
Targeted inhibition of DYRK1A promotes radiosensitivity in pancreatic cancer. (**A**,**B**) Dose–response curves of TB32047 (**A**) and MIA PACA-2 (**B**) wild-type cells to Harmine (**left**). TB32047 and MIA PACA-2 wild-type cells were treated with the indicated concentrations of inhibitors along with different doses of radiation and then subjected to clonogenic assays. Representative images of three independent experiments (*n* = 3) are shown (**middle**). The colony numbers were counted, normalized, and statistically analyzed (**right**). Data are presented as mean ± SD. *, *p* < 0.05; **, *p* < 0.01 and ***, *p* < 0.001 by 2-tailed unpaired Student’s *t* test. (**C**) Immunofluorescence of TB32047 wild-type cells was treated with the indicated concentrations of inhibitors at different time points with 2 Gy irradiation. Representative images of three independent experiments (*n* = 3) are shown. (Blue-DAPI, Green-γH2A.X. Scale bar: white, 20 μm). (**D**) Statistical analysis of γH2A.X foci per cell. Number of foci was counted and normalized, and a minimum of 200 cells were analyzed. *, *p* < 0.05; **, *p* < 0.01; and ****, *p* < 0.0001 by 2-tailed unpaired Student’s *t* test. ns. not significant. (**E**) Western blot of TB32047 and MIA PACA-2 treated with inhibitor alone, X-rays alone, or their combination.

To corroborate the knockout results, we used Harmine, a targeted inhibitor of DYRK1A, and investigated its potential as a radiation sensitizer. We initially constructed dose–response curves in TB32047 and MIA PACA-2 cells, which revealed that Harmine inhibited the ability of PC cells to form living colonies in a dose-dependent manner (Figure 4A,B, left panels). Treatment with non-lethal concentrations of Harmine potentiated the anti-clonogenic effects of radiation therapy in TB32047 and MIA PACA-2 cells (Figure 4A,B, middle and right panels).

To explore the role of Harmine in DNA damage and repair, we assessed the formation of γH2A.X foci by immunofluorescence staining. Interestingly, we observed that, even in the absence of radiation therapy, TB32047 cells exhibited more γH2A.X foci than untreated cells when pre-treated with sublethal doses of Harmine for 24 h (Figure 4C,D). After radiation treatment, Harmine-treated cells had significantly more DSBs than the untreated group 1 h and 4 h post-irradiation, while a significant number of DSBs remained incompletely repaired 24 h after irradiation (Figure 4C,D). Again, the targeted suppression of DYRK1A has been shown to synergize with radiation, increasing DNA double-strand breaks and impairing DNA repair.

To further elucidate the mechanism of Harmine as a radiosensitizer, we first examined the relevant DNA damage repair pathways mediated by ATM and ATR in the presence of Harmine by Western blot. We found that irradiation induced ATM activation, as shown by phosphorylation at residue 1981, which was enhanced in the presence of Harmine (Figure 4E). We further indicated that the level of phosphorylation at the CHK1 ser345 site and the CHK2 Thr68 site was significantly increased upon combination therapy, while phosphorylation at ser296 was decreased (Figure 4E). In addition, RAD51 but not Ku70 expression was reduced after the combination treatment, indicating that Harmine mediates the repression of DNA DSB repair by HR (Figure 4E).

## 4. Discussion

Over the past few decades, radiotherapy has undergone significant development. It has significantly improved the prognosis of patients with lung cancer [30], head and neck squamous cell carcinoma [31], and esophageal cancers [32]. However, due to the existence of inherent and acquired radioresistance, its utility in pancreatic cancer is severely restricted. As such, we sought to use the cutting-edge CRISPR-Cas9 screening approach to unravel the enigma of radioresistance, identify novel target candidates, and provide sufficient preclinical evidence for clinical application in pancreatic cancer. Our results also simultaneously exemplify the possibility of performing CRISPR-Cas9 screens in an in vitro model such as 3D cell culture. In particular, we demonstrated that the sensitivity of 3D cells to radiation is no different from that of 2D cells, despite their growth in matrigel and special culture media [17,33,34]. As a result, we irradiated cells with two distinct growth patterns using a standard radiographic screening approach. The cellular processes they enriched after screening both corroborated the radiation damage response, confirming the reliability of our screening using the 3D model. Interestingly, although more common pathways were investigated in 2D and 3D screens, there were only a few common genes after screening, and the 3D screen was also enriched for cellular response to growth factor stimulus, MAPK cascade, and the activation of MAPK. This may explain the variability between 2D and 3D screens; however, further data are required to substantiate the dependability of 3D screens and explain the variability between different model screens. Here, by implementing a systematic approach of negative selection of CRISPR-Cas9 screening, we identified several kinases, in particular DYRK1A, whose loss of function enhances the radiotherapy effect of PC. We demonstrated that DYRK1A inactivation sensitized PC cells to X-rays, and targeted DYRK1A inhibitor Harmine strongly synergized with radiotherapy, leading to increased cell death and anti-tumor effect. We further revealed that the anti-tumor effect of the combination therapy is associated with an increase in DNA damage and impaired DNA damage homologous repair after radiotherapy, leading to cell death. Overall, our results provide strong mechanistic evidence for combining DYRK1A inhibitors with RT and support the use of CRISPR-Cas9 screening to identify the target of radiotherapy in preclinical models.

Dual-specificity tyrosine phosphorylation-regulated kinase 1A (DYRK1A) is a dual kinase that plays a versatile role in tumorigenesis [35]. An increasing number of studies have revealed that the DYRK protein kinases are critical regulators of cell proliferation and apoptosis [36]. In several human cancer cell lines, DYRK1A has been shown to act as a caspase-9 Thr125 kinase, inhibiting intrinsic apoptotic pathways [37]. The expression and activity of DYRK1A were shown to be elevated in response to toxic stimuli, indicating a putative involvement for DYRK1A in cellular stress response pathways [37,38]. SIRT1, which is a nicotinamide adenosine dinucleotide (NAD)-dependent class 3 histone deacetylase, was found to play a major role in cellular stress [39]. DYRK1A acts as a kinase for SIRT1, activates its deacetylation activity, and further promotes the inhibition of P53, thereby maintaining cancer cell survival under stressful conditions [39]. A comprehensive interaction screening based on proteomics linked DYRK1A to RNF169 and DNA damage response [40]. The results demonstrated that RNF169 is a DYRK1A substrate, and that DYRK1A binding to RNF169 is necessary for the recruitment of DYRK1A to DSB-induced foci [40]. Our results demonstrated that knockout DYRK1A in PC cell lines might result in increased DNA damage and impaired HRR after DSBs, hence decreasing radioresistance in pancreatic cancer. In irradiated cells, γH2A.X foci increased instantaneously and decreased in subsequent DNA repair. We revealed that in knockout cells, both ATM and ATR-mediated DNA damage response mechanisms were considerably active. The phosphorylation of Chk1 (S345), phosphorylation of CHK2 (T68), and sustained increased expression of γH2A.X are evidence of more severe DNA damage in KO cells [30,41].

Currently, the targeted inhibition of DNA damage, DNA DSB repair pathways, cell cycle checkpoints, etc., has become an attractive strategy for reversing resistance to radiotherapy in PC. A large deep whole-genome sequencing redefined the mutational landscape of pancreatic cancer and showed that variation in chromosomal structure was an important mechanism in pancreatic carcinogenesis [12]. Mutations in many other genes involved in DDR, such as ATM, FANCM, XRCC4, and XRCC6 (Ku70), were detected in tumors with an unstable genome or the BRCA mutational signature. Platinum-based treatment, such as FOLFIRINOX, has improved survival in patients with BRCA1 and BRCA2 mutations, according to the available research [13]. Numerous inhibitors have been developed to target DDR and genomic instability mechanisms, such as PARP inhibitors mainly based on mutations in BRCA1 and BRCA2 [15]. Olaparib was the first PARP inhibitor approved by the FDA [42] (December 2014) for cancer therapy, and PARP-1 inhibitors have been identified and tested after clinical trials and their function in enhancing the response of cancers to radiation has been documented [15,43]. Although Harmine has been classified as an antitumor drug for a long period of time, its unknown mechanism of HR regulation and moderate antitumor activity have precluded its further clinical application [44,45]. We demonstrated that inhibiting DYRK1A with Harmine significantly decreased HRR activity, maintained the activation of the DDR pathway, significantly delayed DSB recovery, and resulted in a significant accumulation of DNA damage. Interestingly, previous studies have shown that Harmine alone could also induce DNA damage [44], including DNA DSBs, which is consistent with our findings. Harmine treatments also induced a certain number of γH2A.X foci at low doses, making Harmine a dual-target drug candidate integrating DNA damage and DNA damage homologous repair. Notably, the combination of targeted inhibition of DYRK1A with radiotherapy provided a synergistic therapeutic effect compared to a single treatment modality. Thus, we report a possible new therapeutic option to overcome PDAC radioresistance and further confirm the feasibility of using a combination of CRISPR-Cas9 screening and 3D cell culture to explore new drug targets. Several radiosensitizers have been clinically studied in a variety of malignancies, including CHK1/CHK2 inhibitors, ATM/ATR inhibitors, and PARP inhibitors, but clinical trials of pancreatic cancer-related radiosensitizers are still relatively scarce and most are in the preclinical stage. Our results are also limited to in vitro cellular experiments confirming that the knockout or targeted inhibition of DYRK1A has an efficient radiosensitizing effect. Despite the widespread use of Harmine as a targeted inhibitor of DYRK1A, its further clinical application is limited by its poor specificity and potential side effects, and further in vivo experiments are still needed to further elucidate the specific mechanisms by which Harmine directly induces DNA damage and participates in DDR inhibition [46]. Our results highlight the need for additional clinical trials to evaluate this newly discovered therapeutic combination in the treatment of patients with PDAC and other malignancies.

## 5. Conclusions

In conclusion, we identified DYRK1A as a novel therapeutic target to overcome PC radioresistance by CRISPR-Cas9 loss-of-function with 2D and 3D screens. The depletion of DYRK1A results in an increase in radiation-induced apoptosis and DNA damage in PC cells. DYRK1A contributes significantly to radioresistance via its regulation of the ATM- and ATR-mediated DNA damage response pathways, as well as by its participation in the HRR process. We further investigated Harmine as a promising radiosensitizer for pancreatic cancer. Our findings illuminate the way forward for further clinical translational research into the most effective methods for developing targeted drugs.

## Data Availability

Raw data of the CRISPR/Cas9 screening data are available at the European nucleotide archive with the accession number PRJEB49806.

## Supplementary Materials

The following are available online at https://www.mdpi.com/article/10.3390/cancers14020326/s1: Table S1: CRISPR-Cas9 Radiotherapy Screening Results-2D; Figure S1: Western blot of TB32047 and MIA PaCa-2 cells transfected with DYRK1A sgRANs or non-targeting control; Figure S2: Western blot of TB32047 and MIA PaCa-2 control or DYRK1A KO single clone cells with 0 Gy or 5 Gy irradiation after 24 h; Figure S3. Western blot of TB32047 and MIA PACA-2 treated with inhibitor alone, X-rays alone, or their combination.
