# Correction: Lan et al. CRISPR-Cas9 Screen Identifies DYRK1A as a Target for Radiotherapy Sensitization in Pancreatic Cancer. *Cancers* 2022, *14*, 326

**DOI:** 10.3390/cancers17193181

**Published:** 2025-09-30

**Authors:** Bin Lan, Siyuan Zeng, Shuman Zhang, Xiaofan Ren, Yuming Xing, Isabella Kutschick, Susanne Pfeffer, Benjamin Frey, Nathalie Britzen-Laurent, Robert Grützmann, Nils Cordes, Christian Pilarsky

**Affiliations:** 1 Department of Surgery, Universitätsklinikum Erlangen, Friedrich-Alexander Universität Erlangen-Nürnberg (FAU), 91054 Erlangen, Germany; binlan1991@163.com (B.L.); siyuanzeng2021@163.com (S.Z.); zhang_shu_man@163.com (S.Z.); renxiaofan1013@gmail.com (X.R.); yumingxingjennifer@gmail.com (Y.X.); isabella.kutschick@uk-erlangen.de (I.K.); susanne.pfeffer@uk-erlangen.de (S.P.); nathalie.britzen-laurent@uk-erlangen.de (N.B.-L.); robert.gruetzmann@uk-erlangen.de (R.G.); 2 Translational Radiobiology, Department of Radiation Oncology, Universitätsklinikum Erlangen, Friedrich-Alexander Universität Erlangen-Nürnberg (FAU), 91054 Erlangen, Germany; benjamin.frey@uk-erlangen.de; 3 OncoRay-National Center for Radiation Research in Oncology, Faculty of Medicine Carl Gustav Carus, Technische Universität Dresden, 01307 Dresden, Germany; nils.cordes@oncoray.de; 4 Helmholtz-Zentrum Dresden-Rossendorf, Institute of Radiooncology-OncoRay, 01328 Dresden, Germany; 5 German Cancer Consortium, Partner Site Dresden: German Cancer Research Center, 69120 Heidelberg, Germany; 6 Department of Radiotherapy and Radiation Oncology, University Hospital Carl Gustav Carus, Technische Universität Dresden, 01307 Dresden, Germany

**Error in Figure**

In the original publication [1], there was a mistake in Figure 2C right as published. An error occurred during figure assembly duplicating part of the figure. The corrected Figure 2C right appears below.

The authors apologize for any inconvenience caused and state that the scientific conclusions are unaffected. This correction was approved by the Academic Editor. The original publication has also been updated.

## Figures and Tables

**Figure 2:**
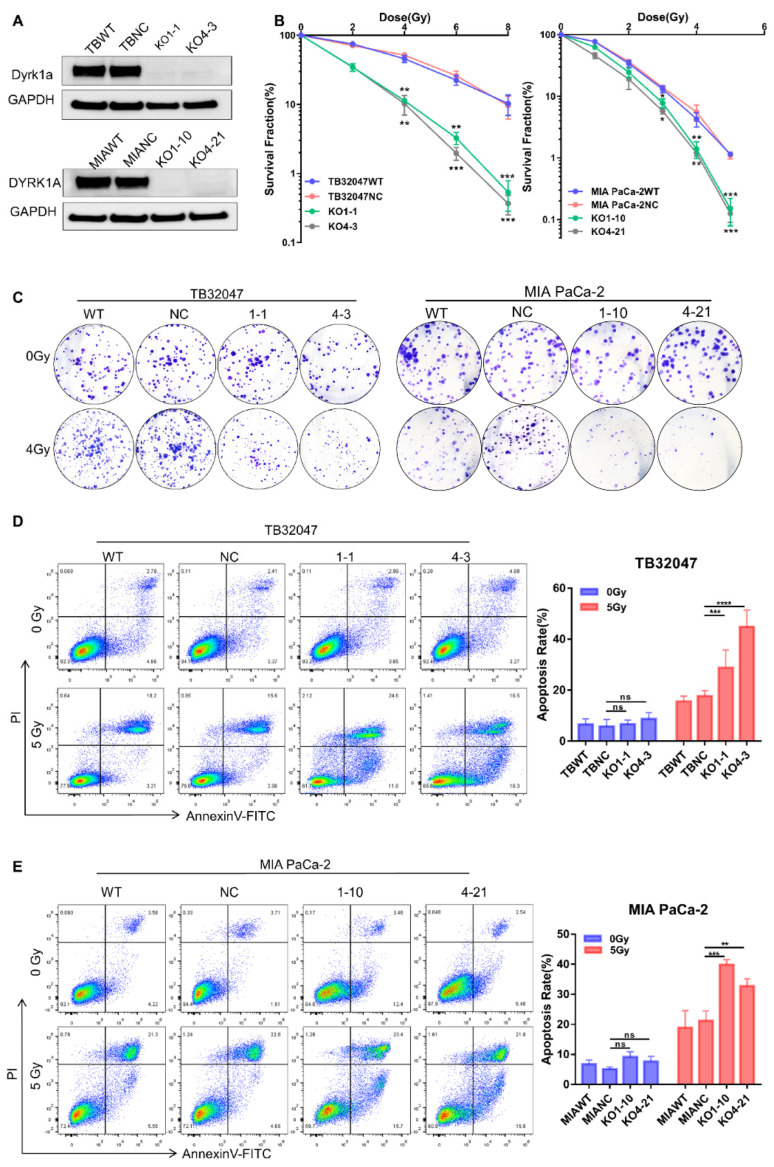
DYRK1A knockout enhances radiotherapy efficacy in pancreatic cancer. (**A**) Western blot of TB32047 and MIA PaCa-2 cells transfected with DYRK1A sgRANs or non-targeting control. (**B**) A clonogenic assay of TB32047 (**left**) and MIA PaCa-2 (**right**) cells with DYRK1A KO and control sgRNA cells was performed after irradiation with different doses of X-rays. The colony numbers were counted and normalized. Data are presented as means of three independent experiments (*n* = 3). *, *p* < 0.05; **, *p* < 0.01; *** and *p* < 0.001 by 2-tailed unpaired Student’s *t* test. (**C**) TB32047 (**left**) and MIA PaCa-2 (**right**) single clones and control cells were treated with different doses of radiation followed by clonogenic assays. Representative images of three independent experiments (*n* = 3) are shown. (**D**,**E**) Flow cytometry-based apoptosis analysis of TB32047 (**D**) and MIA PaCa-2 (**E**) control and DYRK1A KO single clones irradiated with 5 Gy X-rays after 24 h. Representative images of three independent experiments (*n* = 3) and statistical analysis are shown. **, *p* < 0.01; ***, *p* < 0.001 and ****, *p* < 0.0001 by 2-tailed unpaired Student’s *t* test. ns. not significant.
