# Supplementary material for: CRISPR-Cas9 Screen Identifies DYRK1A as a Target for Radiotherapy Sensitization in Pancreatic Cancer
Source: Cancers (Basel). 2022 Jan 10;14(2):326. doi: 10.3390/cancers14020326 (PMC8773906; doi:10.3390/cancers14020326)
Supplement: Supplementary file 1 [file cancers-14-00326-s001.zip › cancers-1531441-supplementary/supplemetary_files_BL_2/cancers-1531441-supplementary figures.pdf]

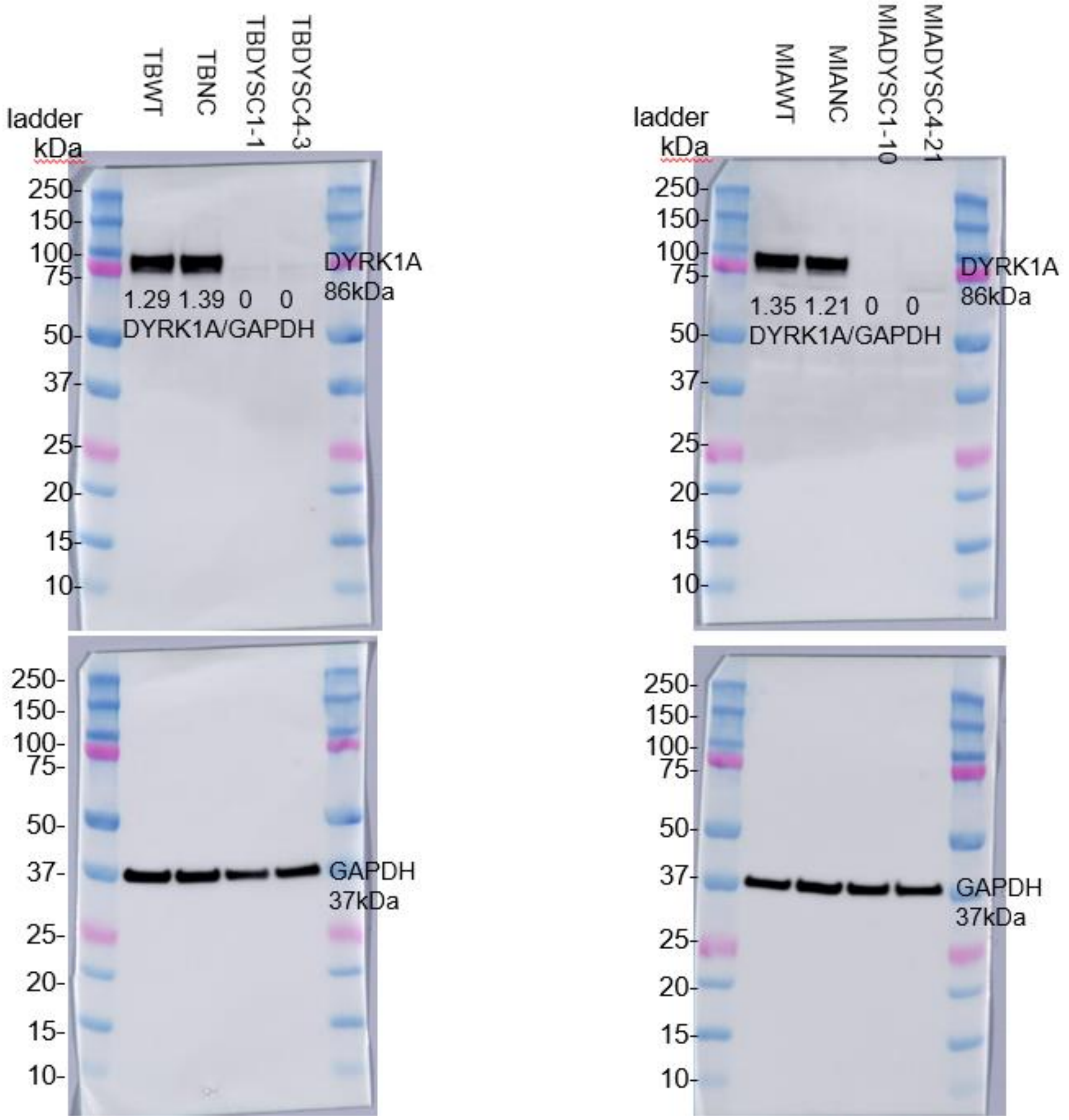

Figure S1. Western blot of TB32047 and MIA PaCa-2 cells transfected with DYRK1A sgRANs or non-targeting control.

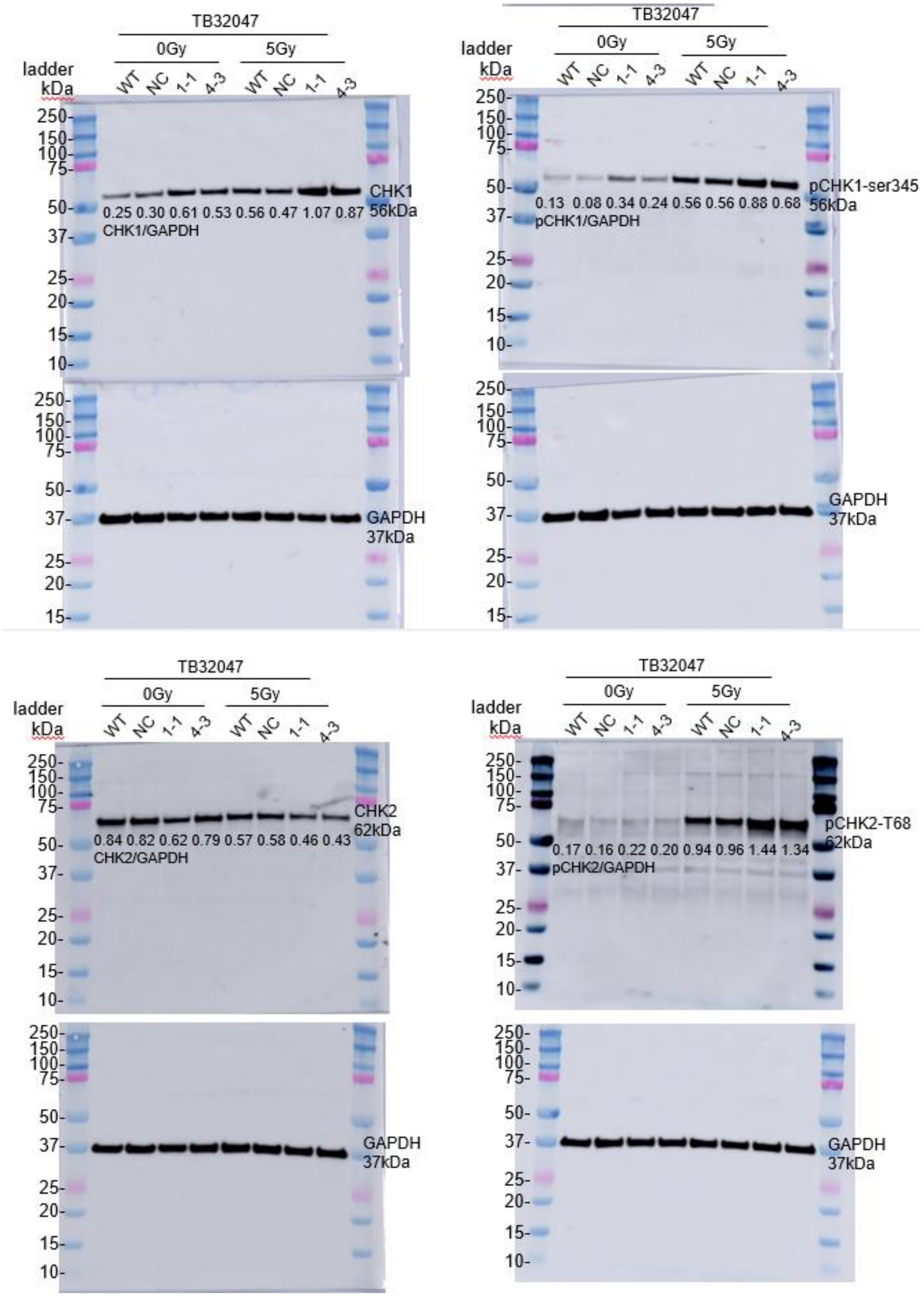
TB32047
0Gy
5Gy
WT NC 1-1 4-3 WT NC 1-1 4-3
ladder
kDa
250- 150- 100- 75- 50- 37- 25- 20- 15- 10-
CHK1
56kDa
0.25 0.30 0.61 0.53 0.56 0.47 1.07 0.87
CHK1/GAPDH
GAPDH
37kDa
TB32047
0Gy
5Gy
WT NC 1-1 4-3 WT NC 1-1 4-3
ladder
kDa
pCHK1-ser345
56kDa
0.13 0.08 0.34 0.24 0.56 0.56 0.88 0.68
pCHK1/GAPDH
GAPDH
37kDa
TB32047
0Gy
5Gy
WT NC 1-1 4-3 WT NC 1-1 4-3
ladder
kDa
CHK2
62kDa
0.84 0.82 0.62 0.79 0.57 0.58 0.46 0.43
CHK2/GAPDH
GAPDH
37kDa
TB32047
0Gy
5Gy
WT NC 1-1 4-3 WT NC 1-1 4-3
ladder
kDa
pCHK2-T68
62kDa
0.17 0.16 0.22 0.20 0.94 0.96 1.44 1.34
pCHK2/GAPDH
GAPDH
37kDa

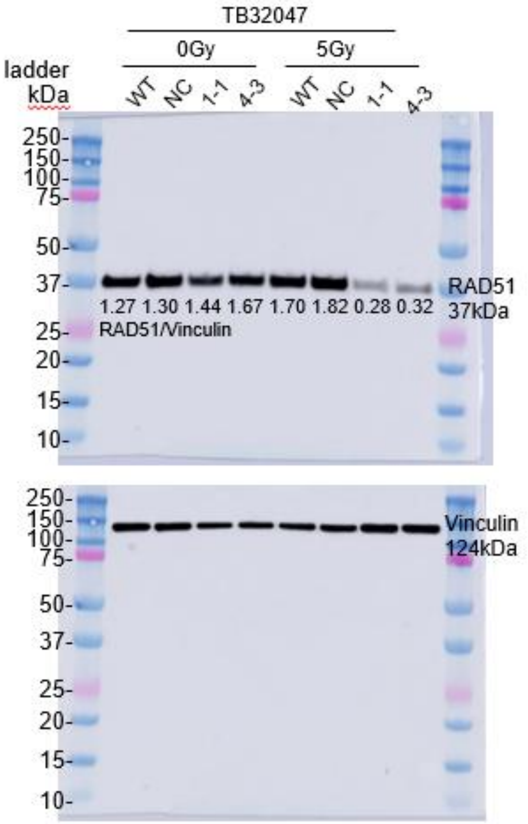
TB32047
0Gy
5Gy
ladder
kDa
WT
NC
1-1
4-3
WT
NC
1-1
4-3
250-
150-
100-
75-
50-
37-
25-
20-
15-
10-
1.27 1.30 1.44 1.67 1.70 1.82 0.28 0.32
RAD51/Vinculin
RAD51
37kDa
Vinculin
124kDa

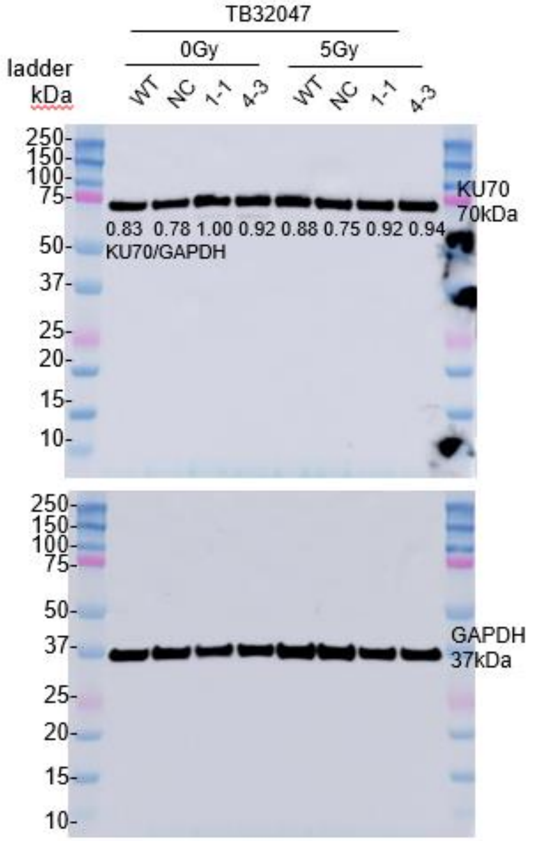
TB32047
0Gy
5Gy
ladder
kDa
WT
NC
1-1
4-3
WT
NC
1-1
4-3
250-
150-
100-
75-
50-
37-
25-
20-
15-
10-
0.83 0.78 1.00 0.92 0.88 0.75 0.92 0.94
KU70/GAPDH
KU70
70kDa
GAPDH
37kDa

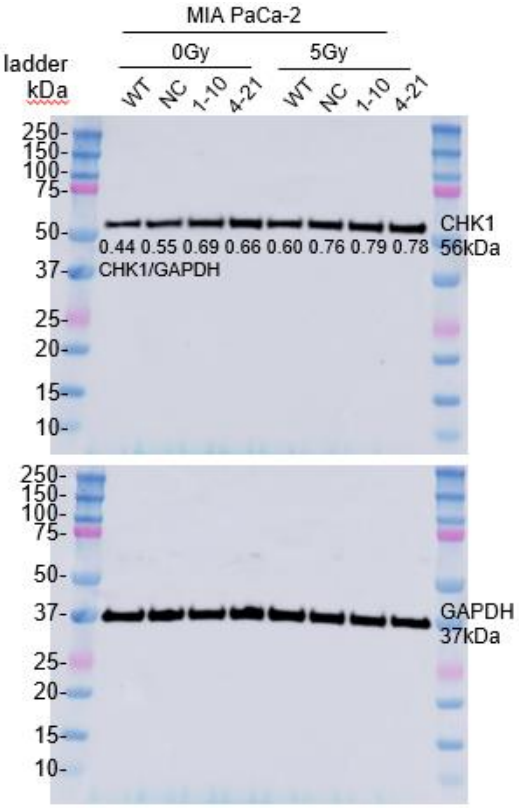
MIA PaCa-2
0Gy
5Gy
ladder
kDa
WT
NC
1-10
4-21
WT
NC
1-10
4-21
250-
150-
100-
75-
50-
37-
25-
20-
15-
10-
0.44 0.55 0.69 0.66 0.60 0.76 0.79 0.78
CHK1/GAPDH
CHK1
56kDa
GAPDH
37kDa

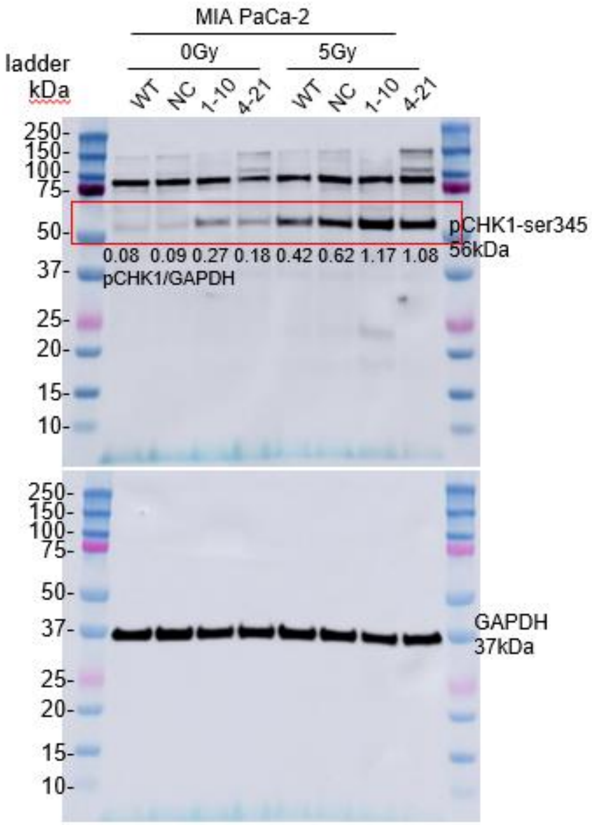
MIA PaCa-2
0Gy
5Gy
ladder
kDa
WT
NC
1-10
4-21
WT
NC
1-10
4-21
250-
150-
100-
75-
50-
37-
25-
20-
15-
10-
0.08 0.09 0.27 0.18 0.42 0.62 1.17 1.08
pCHK1/GAPDH
pCHK1-ser345
56kDa
GAPDH
37kDa

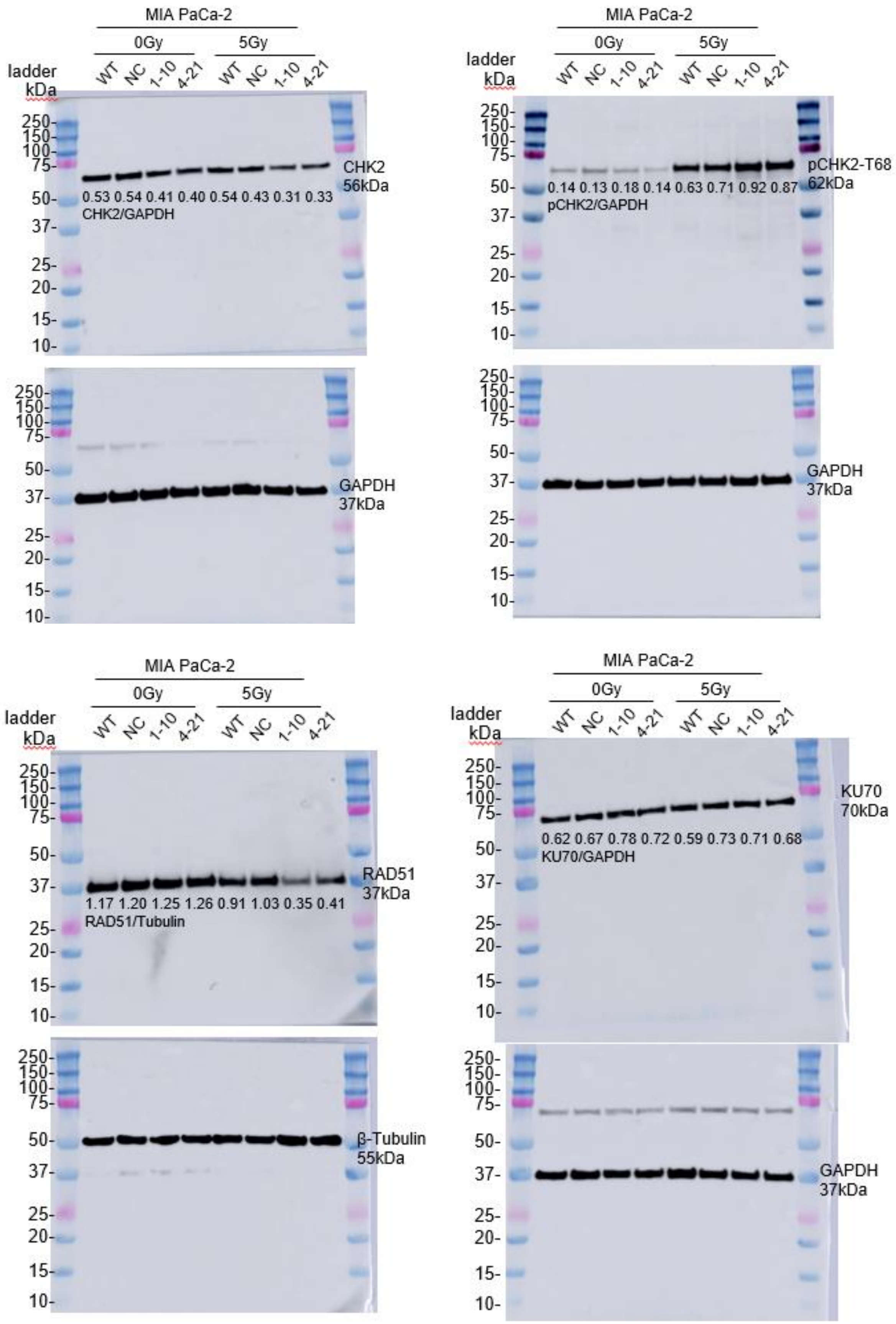

Figure S2. Western blot of TB32047 and MIA PaCa-2 control or DYRK1A KO single clone cells with 0 Gy or 5 Gy irradiation after 24 h.

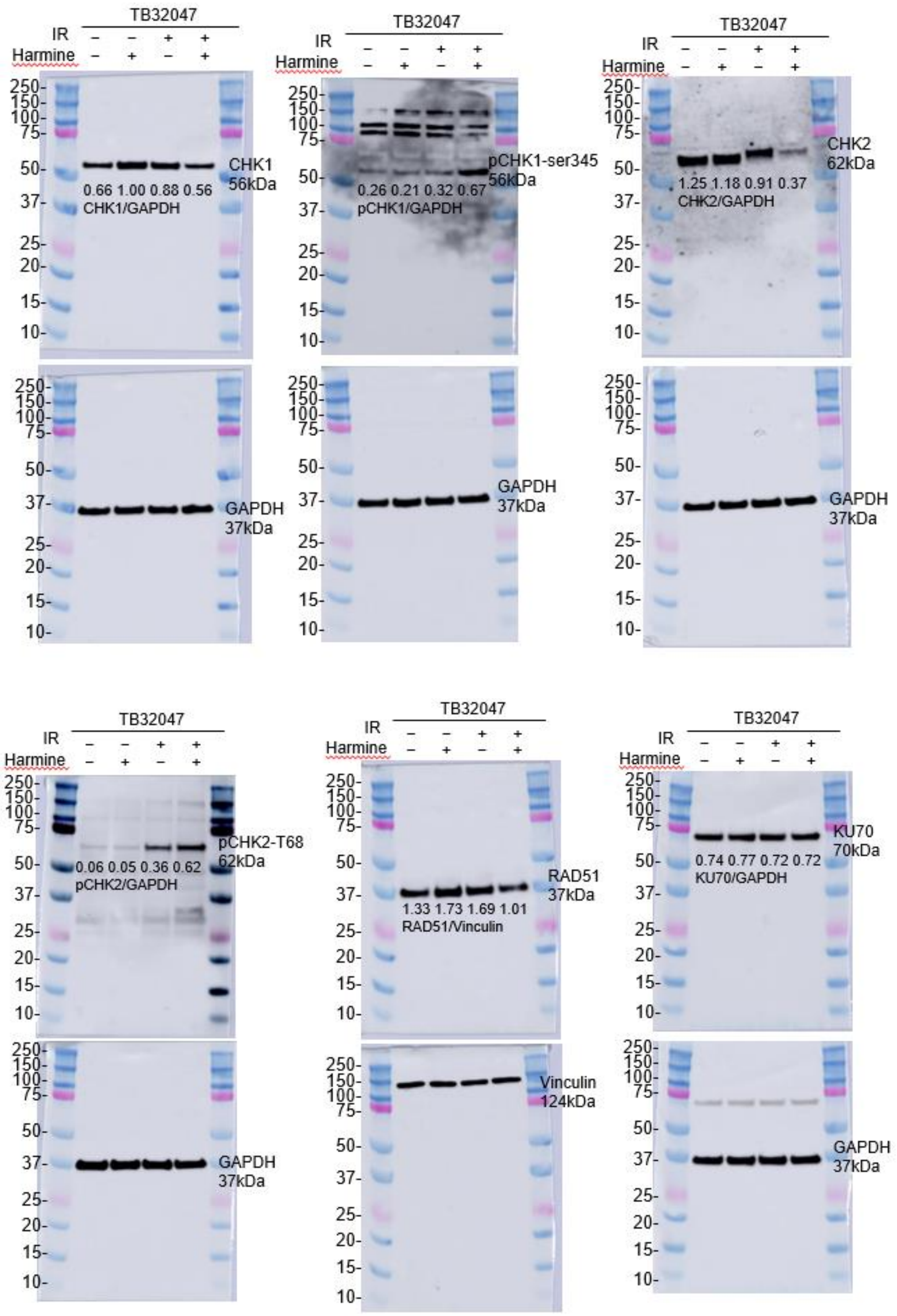
TB32047
IR – – + +
Harmine – + – +
CHK1
56kDa
0.66 1.00 0.88 0.56
CHK1/GAPDH
GAPDH
37kDa
pCHK1-ser345
56kDa
0.26 0.21 0.32 0.67
pCHK1/GAPDH
CHK2
62kDa
1.25 1.18 0.91 0.37
CHK2/GAPDH
pCHK2-T68
62kDa
0.06 0.05 0.36 0.62
pCHK2/GAPDH
RAD51
37kDa
1.33 1.73 1.69 1.01
RAD51/Vinculin
Vinculin
124kDa
KU70
70kDa
0.74 0.77 0.72 0.72
KU70/GAPDH
250-
150-
100-
75-
50-
37-
25-
20-
15-
10-

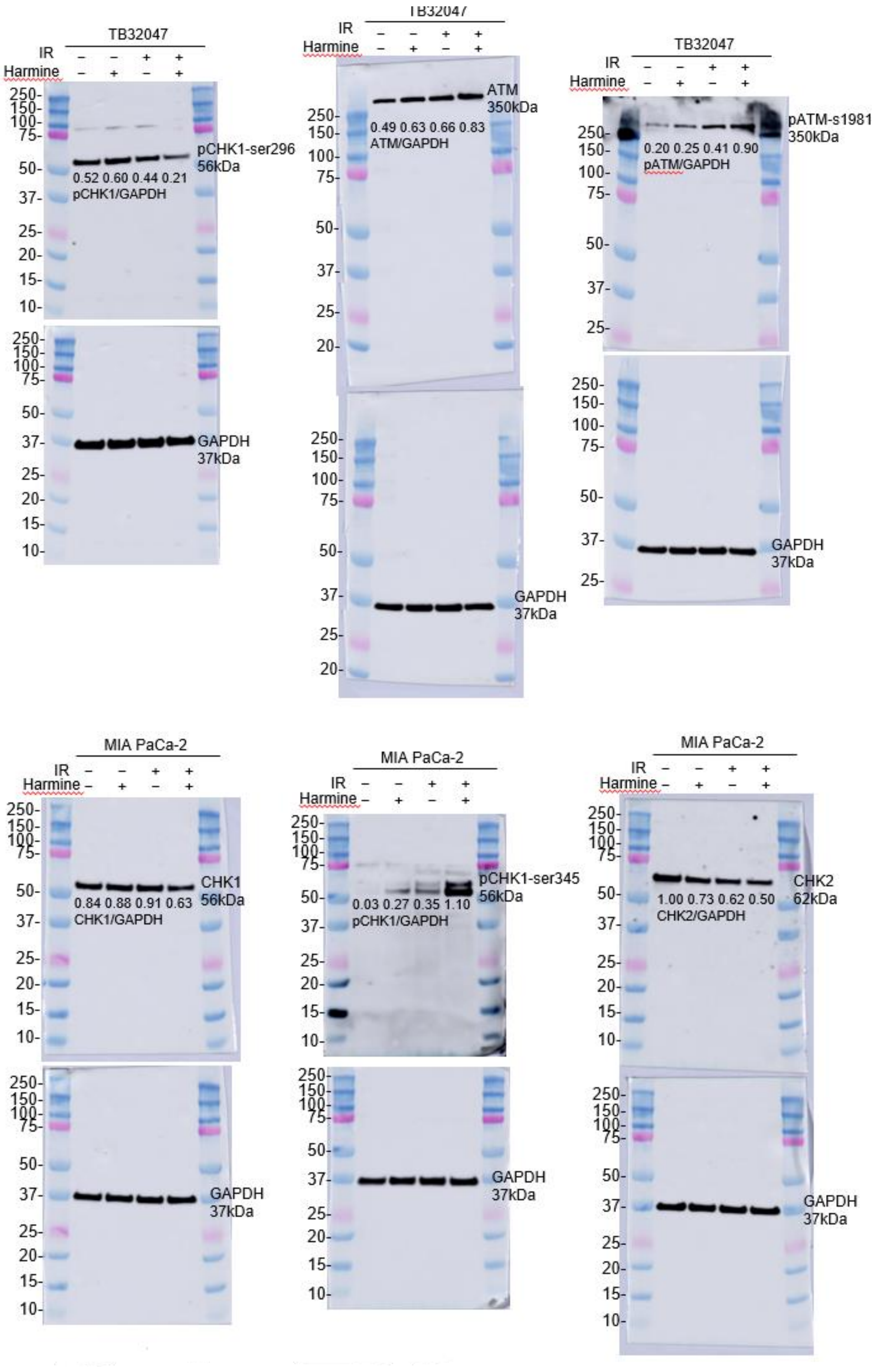
TB32047
IR – – + +
Harmine – + – +
pCHK1-ser296
56kDa
0.52 0.60 0.44 0.21
pCHK1/GAPDH
GAPDH
37kDa
TB32047
IR – – + +
Harmine – + – +
ATM
350kDa
0.49 0.63 0.66 0.83
ATM/GAPDH
GAPDH
37kDa
TB32047
IR – – + +
Harmine – + – +
pATM-s1981
350kDa
0.20 0.25 0.41 0.90
pATM/GAPDH
GAPDH
37kDa
MIA PaCa-2
IR – – + +
Harmine – + – +
CHK1
56kDa
0.84 0.88 0.91 0.63
CHK1/GAPDH
GAPDH
37kDa
MIA PaCa-2
IR – – + +
Harmine – + – +
pCHK1-ser345
56kDa
0.03 0.27 0.35 1.10
pCHK1/GAPDH
GAPDH
37kDa
MIA PaCa-2
IR – – + +
Harmine – + – +
CHK2
62kDa
1.00 0.73 0.62 0.50
CHK2/GAPDH
GAPDH
37kDa
250- 150- 100- 75- 50- 37- 25- 20- 15- 10-

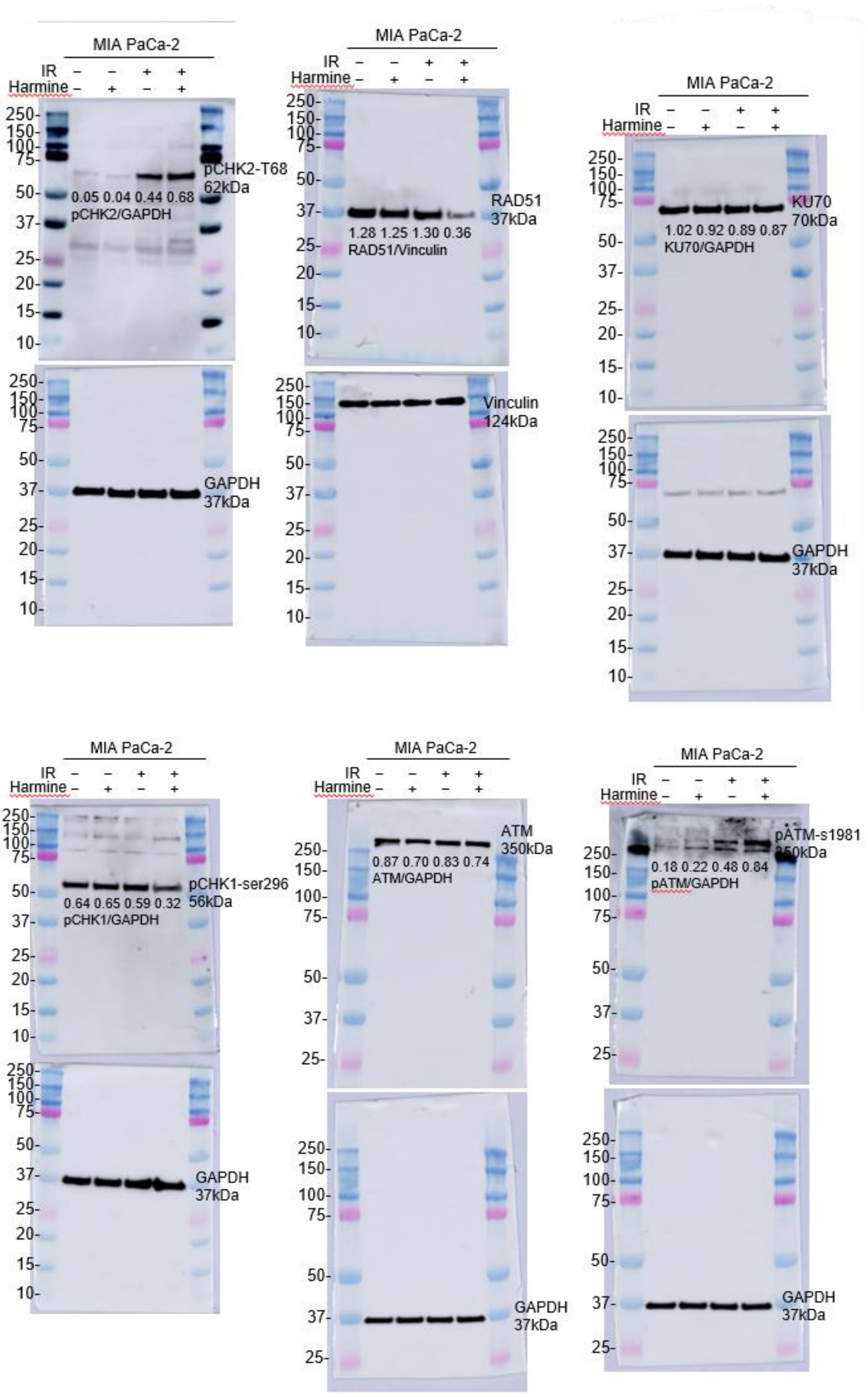
MIA PaCa-2
IR – – + +
Harmine – + – +
pCHK2-T68
62kDa
0.05 0.04 0.44 0.68
pCHK2/GAPDH
GAPDH
37kDa
RAD51
37kDa
1.28 1.25 1.30 0.36
RAD51/Vinculin
Vinculin
124kDa
KU70
70kDa
1.02 0.92 0.89 0.87
KU70/GAPDH
pCHK1-ser296
56kDa
0.64 0.65 0.59 0.32
pCHK1/GAPDH
ATM
350kDa
0.87 0.70 0.83 0.74
ATM/GAPDH
pATM-s1981
350kDa
0.18 0.22 0.48 0.84
pATM/GAPDH
250- 150- 100- 75- 50- 37- 25- 20- 15- 10-

Figure S3. Western blot of TB32047 and MIA PACA-2 treated with inhibitor alone, X-rays alone, or their combination.
